# Nontuberculosis mycobacterial infections at a specialized tuberculosis treatment centre in the Republic of Korea

**DOI:** 10.1186/s12879-017-2532-4

**Published:** 2017-06-15

**Authors:** Hee Jung Yoon, Hwa Young Choi, Moran Ki

**Affiliations:** 1 Division of Infectious Diseases, Seoul Metropolitan Government Seobuk Hospital, Seoul, South Korea; 2 Department of Cancer Control and Population Health, Graduate School of Cancer Science and Policy, National Cancer Center, 323 Ilsan-ro, Ilsandong-gu, Goyang, 10408 South Korea

**Keywords:** Nontuberculous mycobacteria, Epidemiology, Korea, Tuberculosis, Surveillance

## Abstract

**Background:**

The incidence of nontuberculous mycobacteria (NTM) infections is increasing worldwide, however formal evaluations of the epidemiology of NTM infections are limited. Understanding the trends and true prevalence of NTM is a major priority for optimizing infection control programmes and resources. The purpose of this study was to investigate the epidemiology, clinical manifestations, and radiologic findings in NTM-infected patients at specialized Tuberculosis (Tb) treatment centre in South Korea, which is endemic to Tb, and find solutions to control NTM infections.

**Methods:**

A retrospective descriptive study was conducted among patients who were diagnosed with NTM from November 2011 to January 2016 at Seoul Metropolitan Government Seobuk hospital, Korea, using medical records and chest radiography results. Prevalence of NTM using national health insurance data was compared to the prevalence and incidence of Tb using National statistics data.

**Results:**

The age- and sex- adjusted prevalence of NTM infection per 100,000 population increased between 2009 (9.4) and 2016 (36.1). However, the prevalence and incidence of Tb per 100,000 population decreased from 106.5 to 74.4, and 81.2 to 61.8, respectively. In total, 64 patients (37 [57.8%] men) were enrolled in the study. Among 33 (51.6%) patients with slowly growing nontuberculous mycobacteria (SGM) infection, 29 were detected with *Mycobacterium avium* complex (*n* = 13, *M. avium*; *n* = 16, *M. intracellulare*), and 4 with *M. kansasii*. Among 31 (48.4%) patients with rapidly growing nontuberculous mycobacteria (RGM) infection, 27 and 4 patients were detected with *M. abscessus* complex and *M. fortuitum* complex, respectively. RGM patients were more likely to have current Tb (*P =* 0.041), cough (*P* < 0.05), and sputum (*P* < 0.01) than SGM patients in the univariate analysis, but not in the multivariate analysis.

**Conclusion:**

Given the increasing prevalence of NTM infections, precise epidemiological and surveillance data should be obtained by reporting NTM infections to public health authorities. Introducing nucleic acid amplification tests to differentiate between Tb and NTM in smear-positive specimens should be considered.

## Background

The incidence of nontuberculous mycobacteria (NTM) infections is increasing worldwide, including in the United States [1]. It is known that the prevalence of NTM infections increases as the prevalence of tuberculosis (Tb) decreases in western developed countries [2]. Defining the epidemiology of NTM has been more challenging than for Tb. Reporting NTM infections to public health authorities is not mandatory, as opposed to reporting of Tb, in most regions of the world. Therefore, precise epidemiological and surveillance data are lacking [1, 3]. This has limited our knowledge and understanding of the impact of NTM infections on public health [3], leading to human infections by mycobacterial persistence and dissemination within healthcare units [4]. Understanding the trends and true prevalence of NTM is a major priority for optimizing infection control programmes and resources [5]. Therefore, the purpose of this study was to investigate the prevalence of NTM using national health insurance (NHI) data, and the epidemiology, clinical manifestations and radiologic findings in NTM patients in a specialized Tb treatment centre in the Republic of Korea.

## Methods

Seobuk Hospital, run by the Seoul metropolitan government is a 450-bed specialized Tb treatment centre. We retrospectively reviewed the medical records and chest radiography results of 64 patients who were diagnosed with NTM infections from November 2011 to January 2016 at the Seobuk Hospital. Factors that were assessed in this study included patients’ clinical and epidemiologic characteristics. We used Korean data from the Health Insurance Review and Assessment Service (HIRA) [6] to estimate the prevalence NTM between 2009 and 2016 using ICD 10th code (A31). The universal NHI provides high coverage (96.6% in 2010) [7] and is mandatory for all people in South Korea. The remaining 3.4% were covered by a separate program called Medical Aid, which is a public assistance program targeted at poor individuals who are recipients of the National Basic Livelihood Security System in South Korea as a part of the social welfare programs. The Medical Aid program classifies beneficiaries into two categories, type 1 and 2, on the basis of being incapable (those under 18 or over 65 years of age, or disabled) or capable of working, respectively. For the incidence and prevalence of Tb, the numbers of new case and total case notified in a given year stratified by age and sex, were used [8]. All incidence and prevalence rates were standardized for age and sex using 2015 estimated Korea population. Univariate analyses using chi-squared (χ2) tests or independent t-tests were conducted to compare rapidly growing nontuberculous mycobacteria (RGM) and slowly growing nontuberculous mycobacteria (SGM) infections. Multivariate analysis was performed to assess the independence of significant variables in the univariate analysis, using a multiple logistic regression model. A *P*-value <0.05 was considered significant. The SPSS version 20.0 (SPSS Inc., Chicago, IL, USA) statistical software package for Windows was used for all statistical analyses. Ethical approval was obtained from Institutional Review Board of Seobuk Hospital for this study (16805–012-HR).

## Results

**Fig. 1:**
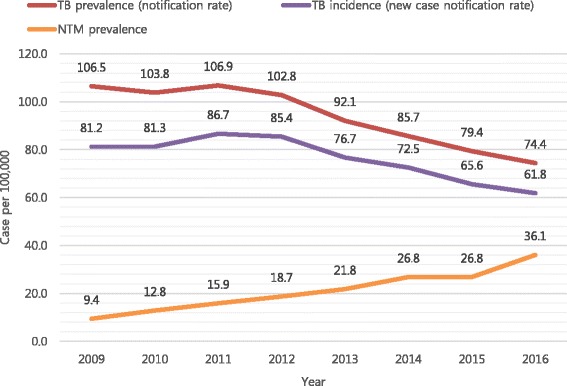
Prevalence and incidence of tuberculosis, and prevalence of nontuberuclous mycobacterial diseases per 100,000 population, 2009–2016, South Korea

The age- and sex- adjusted prevalence of NTM infections per 100,000 population increased between 2009 (9.4) and 2016 (36.1) based on HIRA data [6] (Fig. 1). The age-adjusted prevalence of NTM infections in women was higher than that in men, and the differences increased by year; the prevalence rates for men and women between 2009 and 2016 were 8.6 and 25.3, and 10.3 and 46.8, respectively. However, the prevalence and incidence of Tb per 100,000 population decreased between 2009 and 2016 (106.5 and 74.4 for prevalence; 81.2 and 61.8 for incidence) (Fig. 1).

In total, there were 64 (37 men, 57.8%) NTM patients at the Seobuk hospital from November 2011 to January 2016. The mean age of the patients was 63.5 ±15.6 years. Approximately 40% of patients had initiated anti-Tb medications during the study period within a mean duration of 68 days. Among the 33 (51.6%) SGM patients, 29 and 4 patients had *Mycobacterium (M.) avium* complex (MAC; *n* = 13, *M. avium*; *n* = 16, *M. intracellulare*), and *M. kansasii*, respectively. Among the 31 (48.4%) RGM patients, 27 and 4 patients had *M. abscessus* complex and *M. fortuitum* complex, respectively. Thirty-nine (60.9%) patients had health insurance; 25 had a Medical Aid type 1, and 1 patient had Medical Aid type 2. Forty-two (64.6%) patients resided in Seoul, 19 (29.2%) patients resided in Gyeonggi-do, and 4 (6.2%) patients resided in other provinces. The following chest radiography results were observed: bronchiectasis (*n* = 27, 42.2%), cavities (*n* = 28, 43.8%), destroyed lung (*n* = 12, 18.8%), nodule (*n* = 33, 51.6%), infiltration (*n* = 51, 79.7%), and fibrosis (*n* = 45, 70.3%). Approximately, 15 (23.4%) and 12 (18.8%) had a history of smoking and alcohol consumption, respectively. A history of pulmonary Tb (*n* = 49, 76.6%), recurrent Tb (*n* = 19, 29.7%), and multi-drug resistant Tb (*n* = 9, 14.1%), and current Tb (*n* = 10, 15.6%) were observed. Twenty-five patients (39.1%) initiated anti-Tb medications during the study period, and the mean duration of medication was 68.1±144.6 days [95% CI; 32–103]). The following symptoms were observed: chronic fatigue (*n* = 49, 76.6%), cough (*n* = 61, 95.3%), sputum (*n* = 58, 90.6%), hemoptysis (*n* = 18, 28.1%), dyspnoea on exertion (*n* = 20, 31.3%), fever (*n* = 6, 9.4%), chest pain (*n* = 7, 10.9%), and weight loss (*n* = 17, 26.6%). The mean BMI was 19.7 ± 3.4 kg/m^2^. The mean ± SD of laboratory findings were as follows; haemoglobin 12.3 ± 1.7 g/dL, white blood cell (WBC) count 6961.7 ± 2237.2 cells/mm^3^, protein 7.1 ± 0.7 g/dL, albumin 3.8 ± 0.5 g/dL, aspartate aminotransferase (AST) 24.4 ± 12.3 IU, alanine aminotransferase (ALT) 16.4 ± 14.3 IU, glucose 104.4 ± 24.2 mg/dL, C-reactive protein (CRP) 3.1 ± 3.5 mg/dL, creatinine 0.8 ± 0.2 mg/dL.

According to the univariate analysis, patients with RGM were more likely to have current Tb (*P* = 0.041), cough (*P* < 0.05), and sputum (*P* < 0.01) than patients with SGM (Table 1). When multivariate analysis was performed, these differences were not significant.

**Table 1:** Univariate analysis of slowly and rapidly growing nontuberculous mycobacterial infections among 64 patients in Seobuk hospital, South Korea

Variable	SGM (*n* = 33)	RGM (*n* = 31)	*p*-value
Species	*M.avium* complex 29	*M.abscessus* complex 27	
	*M.avium 13*	*M.fortuitum* complex 4	
	*M.intracellulare 16*		
	*M.kansasii 4*		
Age (years), Mean ± SD	65.5 ± 15.5	61.7 ± 15.9	NS
Sex, *n* (%)			NS
Male	21 (63.6)	16 (51.6)	
Female	12 (36.4)	15 (48.4)	
Chest X-ray, *n* (%)			NS
Bronchiectasis	13 (39.4)	14 (45.2)	
Cavity	11 (33.3)	16 (51.6)	
Destroyed lung	6 (18.2)	6 (19.4)	
Nodule	18 (54.5)	15 (48.4)	
Infiltration	27 (81.8)	24 (77.4)	
Fibrosis	27 (81.8)	18 (58.1)	
Smoking, *n* (%)	7 (21.2)	8 (25.8)	NS
Alcoholic consumption, *n* (%)	4 (12.1)	8 (25.8)	NS
History of pulmonary tuberculosis, *n* (%)	23 (69.7)	26 (83.9)	NS
History of recurrent tuberculosis, *n* (%)	5 (15.2)	14 (45.2)	NS
History of multi-drug resistant tuberculosis, *n* (%)	3 (9.1)	6 (19.4)	NS
Current tuberculosis, *n* (%)	1 (3.0)	9 (29.0)	.041
Symptom screening, *n* (%)			
Chronic Fatigue	26 (78.8)	23 (74.2)	NS
Cough	31 (93.9)	30 (96.8)	<.05
Sputum	29 (87.9)	29 (93.5)	.011
Hemoptysis	8 (24.2)	10 (32.3)	NS
Dyspnea on exertion	11 (33.3)	9 (29.0)	NS
Fever	2 (6.1)	4 (12.9)	NS
Chest pain	3 (9.1)	4 (12.9)	NS
Weight (kg), Mean ± SD	49.1 ± 7.1	52.6 ± 13.5	NS
Hemoglobin (g/dL), Mean ± SD	12.2 ± 1.2	12.4 ± 2.0	NS
WBC (/mm^3^), Mean ± SD	6967.7 ± 2043.6	6867.9 ± 2463.3	NS
Protein (g/dL), Mean ± SD	7.2 ± 0.7	7.0 ± 0.8	NS
Albumin (g/dL), Mean ± SD	3.8 ± 0.5	3.8 ± 0.6	NS
AST (IU), Mean ± SD	24.6 ± 12.5	24.6 ± 12.2	NS
ALT (IU), Mean ± SD	15.2 ± 8.8	17.9 ± 18.8	NS
Glucose (mg/dL), Mean ± SD	107.4 ± 25.3	100.7 ± 23.3	NS
CRP (mg/dL), Mean ± SD	3.3 ± 3.4	3.0 ± 3.8	NS
Creatinine (mg/dL), Mean ± SD	0.8 ± 0.2	0.9 ± 0.3	NS

*SGM* slowly growing nontuberculous mycobacteria; *RGM* rapidly growing nontuberculous mycobacteria, *NS* not statistically significant (*p* > 0.05). *WBC* white blood cell count; *AST* aspartate transaminase; *ALT* alanine transaminase; *CRP* C-reactive protein

## Discussions

In 1987, the US Centers for Disease Control and Prevention (CDC) estimated a population NTM disease rate of 1.8/100,000 between 1981 and 1983 in North America [9]. However, the overall average annual age-adjusted prevalence rate rose from 8.7 to 13.9 per 100,000 persons between the 2008 and 2013 within five states of the United States [10]. The need for updated data was later recognized because of the increasing numbers of published case series, and the widespread belief among clinicians that NTM lung disease was becoming more prevalent. Investigators at a reference hospital in Korea noted an increase in the number of NTM patients from 2002 (*n* = 82) to 2008 (*n* = 133) [1]. Based on the Korean data from NHI, the prevalence of NTM infection per 100,000 population increased from 9.4 in 2009 to 36.1 in 2016 compared to the decreased prevalence of Tb from 106.5 in 2009 to 74.4 in 2016. Possible explanations for the perceived increase in NTM disease include an aging population, increase in the prevalence of immune-modulating co-morbidities (i.e., diabetes mellitus and obstructive pulmonary disease), oesophageal motility disorders, immunosuppressive medication use, and the development of sensitive diagnostic technology [11, 12]. One study showed that localized immunosuppression increased the risk of pulmonary NTM disease in a cohort of 464 patients receiving inhaled corticosteroids for asthma treatment [13]. In a recent systemic review, an increase in the proportion of mycobacterial disease caused by NTM was reported in several settings that had a decrease in Tb prevalence rates [14], as was similar to the findings in our study.

Notification of Tb but not of NTM, is mandatory in Taiwan. Partly due to the strict regulation of Tb notification, several patients infected with NTM were notified as Tb cases (9 per 100,000 patients), imposing additional burdens on the public health system [15]. NTM patients are subjected to mandatory public health reporting in Queensland, Australia, where pulmonary isolates increased from 5.5 to 10.2/100,000 population over a 6-year period [16]. However, NTM reporting is not mandatory in Japan.

The most often isolated cases in the USA are MAC cases, followed by *M. kansasii* and *M. abscessus* [12]. In Japan, MAC accounts for the majority of infections [17]. In this study, the proportion of SGM and RGM cases was 51.6% and 48.4%, respectively. MAC and *M. abscessus* were the most common SGM and RGM infections, respectively. These findings were similar to those from the study by Koh et al. in which *M. abscessus* was found to be the second most common pathogen responsible for lung diseases caused by NTM, after MAC in the Republic of Korea [18, 19]. The identification of bacterial species is clinically important because treatment and response rates differ depending on the bacterial species, and some are refractory disease [20].

Pulmonary presentation of NTM was characterized by the presence of bronchiectasis, cavitation, pneumoconiosis, fibrosis, and nodules. We also observed these radiographic findings, such as bronchiectasis (44.6%), cavities (43.1%), destroyed lung (18.5%), nodules (52.3%), infiltration (78.5%), and fibrosis (70.8%). The cardinal symptoms of fever, chest pain, and unintentional weight loss were prevalent in the survey participants with NTM. We also observed cough (93.8%), sputum (93.8%), chronic fatigue (75.4%), dyspnoea on exertion (30.8%), hemoptysis (27.7%), weight loss (26.2%), chest pain (10.8%), and fever (9.2%) among patients enrolled in this study.

Misdiagnosis or delayed diagnosis of NTM, due to similarity in clinical presentation to Tb, can result in inappropriate isolation with psychosocial stress [15] and serious morbidity and mortality [3]. Traditional culture methods, which take up to 6 weeks, may be required in order to differentiate the Tb and NTM [21]. It will be helpful to introduce nucleic acid amplification (NAA) tests as an additional assay for patients with acid fast bacilli (AFB) smear-positive sputum [22]. NAA tests performed in a timely manner will be helpful in differentiating between Tb and NTM in smear-positive specimens [15]. As our centre is specialized in Tb treatment, many patients initiated anti-Tb medications prior to obtaining their Tb results. Approximately 40% of patients had initiated anti-Tb medications during the study period within a mean duration of 68 days. If the NAA test was conducted when AFB was positive, the rate of misdiagnosis and mistreatment will decrease, further leading to decreased medical expenses, decreased side effects of anti-Tb medication, increased NTM diagnosis, and improvements to the quality of life in patients. National laboratories still need to standardize NTM protocols for improved diagnostic methods and management.

To the best of our knowledge, this was the first study conducted using epidemiologic NTM data from a specialized Tb treatment centre in Korea, a country that is endemic to Tb. As Tb and NTM show similar clinical manifestations, and relatively lower incidence of NTM, NTM has been neglected until now limiting the available epidemiologic data on NTM. Our study highlights the negative impact of NTM on Tb surveillance and control system. Understanding the epidemiology of NTM lung diseases remains crucial to addressing this burgeoning public health challenge [1]. It is essential to ensure that the de-notification of patients infected with NTM occurs as early as possible to reduce the burden on the public health system and ensure the quality of the Tb surveillance system [15]. Finally, a comprehensive multi-country evaluation of NTM is needed to better understand the extent of the NTM burden on the globe and to design strategic action plans.

## Conclusions

Given the increasing incidence of NTM infections worldwide, precise epidemiological and surveillance data should be obtained through the reporting of NTM infections to public health authorities. NAA tests should be considered to differentiate Tb and NTM in smear-positive specimens.

## References

[1] Kendall BA, Winthrop KL (2013). Update on the epidemiology of pulmonary nontuberculous mycobacterial infections. Semin Respir Crit Care Med.

[2] Hoefsloot W (2013). The geographic diversity of nontuberculous mycobacteria isolated from pulmonary samples: an NTM-NET collaborative study. Eur Respir J.

[3] Velayati AA (2015). Nontuberculous mycobacteria in Middle East: current situation and future challenges. Int J Mycobacteriol.

[4] Sousa S (2015). Nontuberculous mycobacteria pathogenesis and biofilm assembly. Int J Mycobacteriol..

[5] Gopinath K, Singh S (2010). Non-tuberculous mycobacteria in TB-endemic countries: are we neglecting the danger. PLoS Negl Trop Dis.

[6] Kim L, Kim JA, Kim S (2014). A guide for the utilization of Health Insurance Review and Assessment Service national patient samples. Epidemiol Health.

[7] Korean Statistical Information Service, National Health Insurance Statistics. 2017 [cited 2017 April 20]; Available from: http://kosis.kr/statisticsList/statisticsList_01List.jsp?vwcd=MT_ZTITLE&parentId=D#SubCon.

[8] Korean Statistical Information Service, Tuberculosis Statistics. [cited. April, 20. 2017; Available from: http://kosis.kr/statisticsList/statisticsList_01List.jsp?vwcd=MT_ZTITLE&parmTabId=M_01_01#SubCont.

[9] O'Brien RJ, Geiter LJ, Snider DE (1987). The epidemiology of nontuberculous mycobacterial diseases in the United States: results from a National Survey 1. Am Rev Respir Dis.

[10] Donohue MJ, Wymer L (2016). Increasing prevalence rate of nontuberculous mycobacteria infections in five states, 2008-2013. Ann Am Thorac Soc.

[11] Winthrop KL (2010). Pulmonary disease due to nontuberculous mycobacteria: an epidemiologist’s view. Future Microbiol.

[12] Griffith DE (2007). An official ATS/IDSA statement: diagnosis, treatment, and prevention of nontuberculous mycobacterial diseases. Am J Respir Crit Care Med.

[13] Hojo M (2012). Increased risk of nontuberculous mycobacterial infection in asthmatic patients using long-term inhaled corticosteroid therapy. Respirology.

[14] Brode S, Daley C, Marras T (2014). The epidemiologic relationship between tuberculosis and non-tuberculous mycobacterial disease: a systematic review. The International Journal of Tuberculosis and Lung Disease.

[15] Chiang CY (2015). Surveillance of tuberculosis in Taipei: the influence of nontuberculous mycobacteria. PLoS One.

[16] Thomson RM (2010). N.T.M.W.G.A.Q.T.C. Centre, and L. Queensland mycobacterial reference, changing epidemiology of pulmonary nontuberculous mycobacteria infections. Emerg Infect Dis.

[17] Ito Y (2015). Increasing patients with pulmonary Mycobacterium Avium Complex disease and associated underlying diseases in Japan. J Infect Chemother.

[18] Koh WJ (2006). Clinical significance of nontuberculous mycobacteria isolated from respiratory specimens in Korea. Chest.

[19] Ryoo SW (2008). Spread of nontuberculous mycobacteria from 1993 to 2006 in Koreans. J Clin Lab Anal.

[20] Kodana M (2016). Utility of the MALDI-TOF MS method to identify nontuberculous mycobacteria. J Infect Chemother.

[21] Control, C.f.D. and Prevention, Updated guidelines for the use of nucleic acid amplification tests in the diagnosis of tuberculosis. MMWR. Morbidity and mortality weekly report. 2009; **58**(1): p. 7. 19145221

[22] Lai CC (2012). Nontuberculous mycobacterial infections in cancer patients in a medical center in Taiwan, 2005-2008. Diagn Microbiol Infect Dis.

